# Skin Care Product Rich in Antioxidants and Anti-Inflammatory Natural Compounds Reduces Itching and Inflammation in the Skin of Atopic Dermatitis Patients

**DOI:** 10.3390/antiox11061071

**Published:** 2022-05-28

**Authors:** Yu Zhang, Nina Heinemann, Franziska Rademacher, Maxim E. Darvin, Christian Raab, Cornelia M. Keck, Henning Vollert, Joachim W. Fluhr, Regine Gläser, Jürgen Harder, Martina C. Meinke

**Affiliations:** 1 Department of Dermatology, Venerology and Allergology, Charité-Universitätsmedizin Berlin, Corporate Member of Freie Universität Berlin, Humboldt-Universität zu Berlin, and Berlin Institute of Health, Charitéplatz 1, 10117 Berlin, Germany; yu.zhang@charite.de (Y.Z.); maxim.darvin@charite.de (M.E.D.); christian.raab@pharmazie.uni-marburg.de (C.R.); joachim.fluhr@charite.de (J.W.F.); 2 Department of Dermatology, Medical Faculty, Christian-Albrecht University Kiel, Arnold-Heller-Str. 3, 24105 Kiel, Germany; nheinemann@dermatology.uni-kiel.de (N.H.); frademacher@dermatology.uni-kiel.de (F.R.); rglaeser@dermatology.uni-kiel.de (R.G.); jharder@dermatology.uni-kiel.de (J.H.); 3 Department of Pharmaceutics and Biopharmaceutics, Philipps-Universität Marburg, Robert-Koch-Str. 4, 35037 Marburg, Germany; cornelia.keck@pharmazie.uni-marburg.de; 4 Bioactive Food GmbH, 23795 Bad Segeberg, Germany; henning_vollert@t-online.de; 5 Institute of Allergy, Charité-Universitätsmedizin Berlin, Corporate Member of Freie Universität Berlin, Humboldt-Universität zu Berlin, and Berlin Institute of Health, Hindenburgdamm 30, 12203 Berlin, Germany

**Keywords:** atopic dermatitis, green tea, apple extract, Raman spectroscopy, stratum corneum

## Abstract

The atopic dermatitis (AD) complex pathogenesis mechanism reveals marked changes of certain signaling factors as well as some morphological alterations in the epidermis. Reduced resilience against environmental factors and oxidative stress often makes the treatment with corticosteroids or tacrolismus ointments indispensable. In view of the correlation between oxidative stress and AD pathological factors, antioxidants can be incorporated into AD management strategies. This study investigates a curly kale, apple and green tea-containing natural extract rich in antioxidants for its effects on signaling inflammatory molecules and skin barrier enhancement in human epidermal keratinocytes- (NHEKs) based cell assays. Furthermore, the skin penetration on porcine ears was measured ex vivo using Raman micro spectroscopy. Finally, in a double-blind half-side, placebo-controlled clinical study, the effects of a formulation containing this extract were analyzed for the influence of lesion severity, epidermal barrier function, and pruritus in mild to moderately AD patients. Summarizing our results: The extract reduces expression of inflammatory cytokines in keratinocytes and increases barrier-related molecules. The verum formulation with a very high antioxidant capacity used in AD patients with mild to moderate lesions reduces itching, local SCORAD, and improves barrier function and the hydration of skin lesions.

## 1. Introduction

Atopic dermatitis (AD) is a common inflammatory skin disease characterized by epidermal dysfunction. As a common disease with high prevalence on a global scale, it has a significant impact on patients’ quality of life [1]. The pathogenesis of AD is complex, resulting from an interaction of genetic, immunologic and environmental factors [2].

The human epidermis includes the following main layers: basal layer on the basement membrane, spinous, granular and lucidum layers, and the outermost cornified layer—stratum corneum. The stratum lucidum is usually found in areas of thick skin, i.e., the hands, palms, and the soles of the feet. Keratinocytes are the most numerous cells in the epidermis, which are continuously differentiated from the basal layer towards the stratum corneum. Different layers together constitute the multi-tiered barrier of human skin to prevent the loss of water and the infiltration of environmental allergens and microbial pathogens.

Upon terminal differentiation, keratinocytes synthesize several of the barrier-related molecules, including keratin-1 and -10, loricrin, filaggrin, and involucrin [3], hydrolytic enzymes, phospholipids, ceramides, glycosyl ceramides and sterols [4].

These molecules play an important role in maintaining the integrity of the skin’s barrier function.

In AD patients, it can be observed that the expression of terminal differentiation molecules, such as filaggrin, involucrin and loricrin, is reduced. This downregulation is one of the influencing factors that cause skin barrier dysfunction [5]. Loss-of-function mutations in filaggrin have been identified as important genetic determinants for AD development [6,7].

In addition to defects in terminal epithelial differentiation such as lack of filaggrin, immune dysregulation, which is mainly a type 2 response, is also one of the factors leading to skin barrier dysfunction [2]. Skin barrier dysfunction, scratching, bacterial colonization and other factors inducing a type 2 immune response leading to the overexpression of IL-13 and IL-4 produced by T-helper 2(Th2) lymphocytes. IL-13 and IL-4 inhibit the expression of filaggrin by up-regulating the expression of IL-24, increasing the production of chemokines, such as CCL17, CCL22 and CCL26, magnifying IL-31-induced pruritus and down-regulating the expression of antimicrobial peptides [2,8].

Oxidative stress, which refers to the generation of reactive oxygen species (ROS) that exceed the scavenging capacity of the antioxidant system (AOS), plays a crucial role in chronic inflammatory skin diseases such as AD [9]. In AD, oxidative stress caused by Th2 cytokines leads to sustained STAT6 signaling, thus further aggravating the disease symptoms [10].

The basic management strategy of AD is to improve symptoms and establish long-term disease control [11], including reducing acute inflammatory symptoms, restoring skin barrier homeostasis, and avoiding related factors that trigger or aggravate the disease [12].

For most patients with mild AD, topical therapy can control skin inflammation, reduce symptoms and prevent flares [13]. Topical corticosteroids and topical calcineurin inhibitors are first-line treatments for anti-inflammatory and flare reduction; however, they are generally not recommended for long-term daily use due to potential local side effects [14,15,16,17]. Thus, despite the current availability of topical treatments for AD, a new topical formulation is required that could enable a balance between efficacy and safety.

In this study, firstly, the anti-inflammatory and barrier enhancement effects of a new extract were studied in cell culture. The radical scavenging activity was investigated for the extract incorporated into a DAC (Deutscher Arzneimittel-Codex)-base cream directly. Subsequently, the skin penetration efficiency of the cream was studied in ex vivo pig ear skin to ensure the uptake of the formulation.

Finally, in a double-blind half-side, placebo-controlled human in vivo study, we investigated the effects of a formulation containing this extract regarding reducing the lesion severity, improvement of epidermal barrier function, and pruritus relief in mild to moderately AD patients.

## 2. Materials and Methods

### 2.1. Materials

The extract was obtained from Bioactive Food GmbH (Bad Segeberg, Germany) and contained equal amounts of curly kale extract (Anklam Extract GmbH, Anklam, Germany), green tea extract (Eurochem Feinchemie GmbH, Gröbenzell, Germany), apple extract (Herbstreith & Fox KG, Neuenbürg/Württ, Germany) and l-arginin (Roth, Karlsruhe, Germany). The flavonoid extracts were prepared using a polymeric adsorbent (Amberlite XAD) according to the study by Zessner et al. [18].

Within the project several commercially available plant extracts (food grade) were preselected based on published data indicating potential beneficial effects on the skin barrier. Kaempferol, a natural flavonol present in various edible plants (including kale), has been reported to possess various anti-inflammatory properties [19]. Kaempferol reduced gene expression of major proinflammatory cytokines, including interleukin (IL)-6, IL-17A and tumor necrosis factor (TNF)-α, in the psoriatic skin lesion [20]. However, it is unknown whether kaempferol as well as kaempferol flavonoids would have an effect in AD. Various biological activities of catechins, the main constituent of green tea, including antioxidant and anti-inflammatory properties, have been reported [21,22]. Furthermore, apple flavonoids like phlorizin were effective in protecting the skin against UVB-induced skin damage by decreasing ROS overproduction, Cox-2 expression and the subsequent excessive inflammation reactions [23]. L-arginine was added to improve the skin barrier factors and moisten the skin in vivo.

For the ex vivo and in vivo experiments, creams that contained 2% (*w*/*w*) extract in base cream DAC (verum) were produced (Löwen-Apotheke, Bad Segeberg, Germany), and cream base without extract was used as control (placebo). Table 1 provides an overview of the formulations used in this study.

### 2.2. In Vitro Experiments on Cell Culture

Pooled primary normal human epidermal keratinocytes (NHEKs), isolated from the epidermis of juvenile foreskin of three male caucasian donors, were used for the in vitro experiments (Lot Number: 456Z001.1, PromoCell, Heidelberg). NHEKs at passage 4 were seeded in a 24-well plate in Keratinocyte Growth Medium 2 (KGM2) (PromoCell, Heidelberg, Germany) supplemented with KGM2 Supplement Mix including CaCl_2_ (PromoCell). After reaching 90–100% confluency, cells were incubated in a medium containing 1.3 mM CaCl_2_ for 48 h to induce differentiation. Differentiated NHEKs were stimulated with a cytokine mix (IL-22 (10 ng/mL), tumor necrosis factor (TNF) alpha (10 ng/mL), IL-13 (10 ng/mL) and IL-4 (10 ng/mL)) to mimic an AD-like inflammation [24]. Simultaneously, these cells were incubated with a 1:800 dilution of the plant extract mixture (10 mg/mL kale extract, 10 mg/mL green tea extract, 10 mg/mL apple extract, 10 mg/mL l-arginine in 50% ethanol) or vehicle control (50% ethanol) for 20 h at 37 °C in 5% CO_2_ atmosphere.

#### Real-Time PCR

After stimulation, the gene expression levels of AD-relevant genes were determined by real-time PCR. Therefore, total RNA was isolated using the Crystal RNAmagic kit (Biolabproducts, Bebensee, Germany). Isolated RNA was transcribed to cDNA using a PrimeScript reverse transcriptase kit (Takara Bio, Saint-Germain-en-Laye, France). Subsequent analysis by real-time PCR was performed with the QuantStudio3 System (Thermo Fisher Scientific, Schwerte, Germany) using the temperature profile as described previously [25]. Gene expression levels of carbonic anhydrase II (CA2, gene acc. no.: NM_000067.2), CC-chemokine ligand 26 (CCL26, gene acc. no.: NM_006072.4 ), collagen type I alpha 1 chain (COL1A1, gene acc. no.: NM_000088.3), filaggrin (FLG, gene acc. no.: NM_002016.1), Interleukin-24 (IL-24, gene acc. no.: NM_006850.3), involucrin (IVL, gene acc. no.: NM_005547.2), loricrin (LOR, gene acc. no.: NM_000427.2) and homo sapiens ribosomal protein L38 (RPL38, acc. no.: NM_000999.3) were measured. The following intron-spanning primers were used (Table 2):

The gene expression levels were normalized to the constitutively expressed ribosomal protein L38 (RPL38) gene.

### 2.3. Radical Protection Factor (RPF)

The RPF technology can determine the antioxidant capacity of the sample by measuring the free radical scavenging activity [26]. Thus, we used electron paramagnetic resonance (EPR) spectroscopy (X-band EPR spectrometer (9.4 GHz) MiniScope MS5000, Magnettech, Freiberg Instruments, Freiberg, Germany) to determine the RPF of the formulation by measuring the scavenged marker radicals. In this study, the following EPR settings were used: modulation amplitude 2 G, microwave (MW) attenuation 15 dB (MW power 3.16 mW), sweep time 20 s, sweep 95 G, B0-field 3350 G, step 1024 and number(pass) 1. 2,2-Diphenyl-1- picrylhydrazyl (DPPH, Sigma-Aldrich, Steinheim, Germany) was used as a test radical. The signal intensity in the sample is determined by the concentration of DPPH. The lower the DPPH concentration, the weaker the signal and the more active the radical-scavenging activity of the cream. For the measurements, 50 mg of each formulation was diluted in 10 mL of ethanol, and then 400 μL of this solution was mixed with 400 μL DPPH (1 mM). The samples were kept under constant agitation in the dark at room temperature until the end of the measurements. The measurements started directly after mixing with DPPH, and measurements were carried out every hour until the DPPH signal was stable (approx. 19 h). After measuring the EPR signal strength, we used the following formula to calculate RPF:

$$RPF=\frac{RC\;\;RF\;}{PI}$$

Whereby RC is the concentration of the test radical, which was measured in radicals per milliliter, RF is the reduction factor, which represents the difference between the untreated test radical intensity and the decreased signal intensity after treatment with the antioxidant normalized to the signal of the untreated test radical, and PI is the product input, which represents the amount of the substance/product measured in milligrams per milliliter [27]. A positive number N that presents the measuring unit 10^14^ radicals/mg expresses the RPF.

### 2.4. Ex Vivo Investigations on Porcine Ear Skin

Nine fresh porcine ears obtained from the local slaughterhouse were gently washed with running room temperature water and dried with soft paper towels. The hair was gently cut without damaging the stratum corneum. A marker was used to label 1 cm × 2 cm, subsequently, two creams (verum and placebo creams) were applied at two different doses (1 mg/cm^2^ and 2 mg/cm^2^) evenly on the marked areas. The treated porcine ears were left at 32 °C and 90% humidity for 2 h and 4 h, respectively, for passive penetration. Subsequently, confocal Raman micro spectroscopy (CRM) was used to observe the 10 processed skin samples. At least eight points on each skin area without any hair or furrows were selected for CRM measurement and subsequent analysis.

### 2.5. Confocal Raman Microspectroscopy

An in vivo confocal Raman microscope (Model 3510 SCA, RiverD International B.V., Rotterdam, The Netherlands) with an excitation wavelength of 785 nm was applied to the untreated and cream-treated skin. The fingerprint region (400–2000 cm^–1^) was used to analyze the samples, and the Raman spectra were recorded from the skin surface down to a depth of 40 μm at increments of 2 μm. The acquisition time for one spectrum was 5 s, and the maximal laser power on the skin was 20 mW. The Raman spectra were analyzed using the non-restricted multiple least square fit method available in the ‘Skin Tools 2.0’ software developed by RiverD International B.V. [28], and the penetration profiles of the semiquantitative concentration of creams were determined similar to that presented in the literature [29]. The penetration depth value was determined as an intersection of cream-treated and untreated profiles (both calculated using ‘SkinTools’ for certain cream), similar to that used in [30]. The utilized CRM system was described in detail elsewhere [31].

### 2.6. Volunteers and Study Design

Ten volunteers with mild to moderately severe AD according to the SCORAD scale (<50) [32] aged 18–60 years (seven female and three male; median age 33 years) were enrolled in the study. Positive votes for the experiments have been obtained from the ethics committee of the Charité – Universitätsmedizin Berlin (EA1/207/20), which were conducted according to the Declaration of Helsinki as revised in 2013, and informed consent was obtained from all patients.

Key exclusion criteria included: (i) patients with severely infected lesions in need of oral or intravenous antibiotics and auxiliary therapy; (ii) patients with dermatological diagnoses other than AD; (iii) people who cannot take responsibility for themselves; (iv) pregnancy or lactation.

The study was a four-week, randomized, double-blind, controlled half-side comparison study. The symmetrical parts (e.g., bilateral forearms) of each volunteer were randomized for treatment with verum or placebo cream treatment in the morning and evening for four weeks. Both creams were packaged in the same containers and were coded (A or B) by the pharmacist.

Only one of the two creams contained AO extract. No other moisturizers or topical treatments were used during the study to control variables. Evaluation of the outcome parameters was performed at baseline (D0) and after four weeks (D28).

### 2.7. Clinical Assessment

The clinical severity scoring was performed by the SCORAD intensity parameters in the test area (local SCORAD, ranges from 0 to 18), includes the assessments of erythema, edema/papulation, oozing/crusts, excoriation, lichenification, dryness on a 0–3 scale (none-0, mild-1, moderate-2 or severe-3) [33].

The itch/sleeplessness intensity was assessed by the volunteers using a visual analogue scale from 0 (no perceptible itch/sleeplessness) to 10 (worst imaginable itch/sleeplessness) [33].

Transepidermal water loss (TEWL), stratum corneum hydration (capacitance) and erythema were measured using the Tewameter TM 300, the Corneometer CM825 and the Mexameter MX 18 (Courage & Khazaka Electronic GmbH, Cologne, Germany), respectively. None of the patients used any topical skin care product on the test areas at least 24 h before the first test and 12 h before the second. Measurements were taken on the test area of the patients’ lesions after 15 min of inactivity and under controlled environmental conditions (room temperature 20–25 °C; relative humidity 40–60%) [34,35].

The assessment result of erythema and capacitance was based on the average value of three measurements on the test area by the same person. The result of TEWL assessment was the average value of 20 consecutive measurements.

### 2.8. Data Analysis

GraphPad Prism (Version 8) was used for statistical analysis of the in vitro data. Normality was tested by D’Agostino & Pearson omnibus normality test. For data deviating from normality, the non-parametric Kruskal-Wallis test with subsequent Dunn’s Multiple Comparison test was used to determine significant differences.

If the data passed normality but did not have equal variances, a Welch’s-ANOVA with subsequent Dunett´s T3 Multiple comparison test was used to determine significant differences. *p*-values were marked by asterisks: * *p* < 0.05, ** *p* < 0.01.

For the in vivo investigations intra-group comparisons were obtained by the Wilcoxon signed ranks test, and inter-group comparisons were performed using the Mann–Whitney U-test. Calculations were done using SPSS 25. Differences with *p*-values of less than 0.05 were considered statistically significant.

## 3. Results

### 3.1. Radical Scavenging Activity of the Formulations

The RPF for verum, determined by EPR spectroscopy, was (690 ± 30) × 10^14^ radicals/mg, while the RPF for placebo was almost 0. This proves that verum has very high radical scavenging activity, indicating high antioxidant properties.

### 3.2. Beneficial Effects of the Extract in an In Vitro AD Cell Culture Model

**Figure 1:**
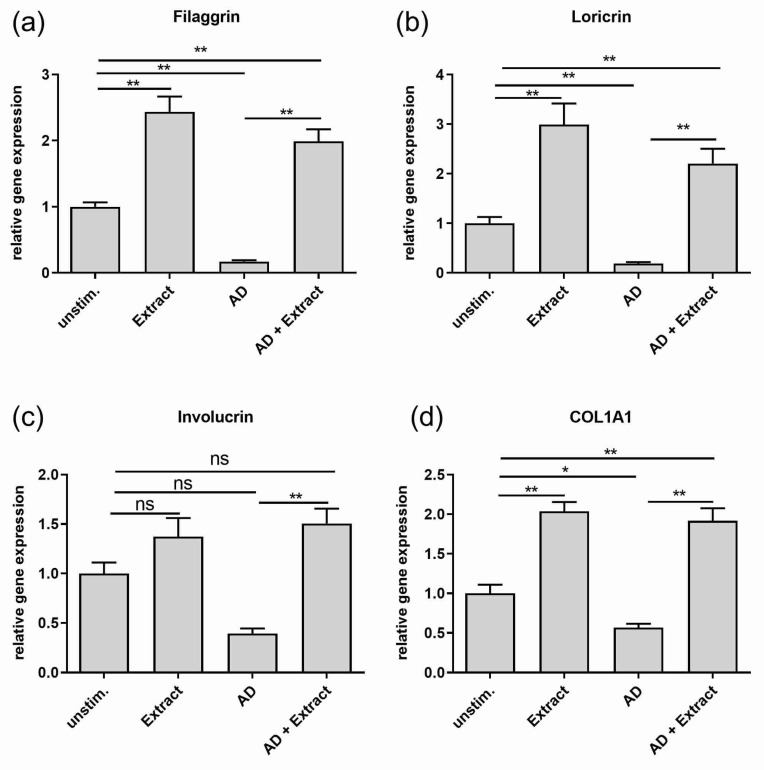
Effect of the plant extract mixture on the gene expression of skin barrier and extracellular matrix molecules in an AD-like in vitro model. NHEKs not treated with specific cytokines were used to reflect healthy skin conditions. These cells were left unstimulated (unstim.) or stimulated with the plant extract (Extract) for 20 h. NHEKs were also treated for 20 h with an AD cytokine mix (IL-22, TNF-alpha, IL-13 and IL-4 (each 10 ng/mL)) either in the absence (AD) or in the presence of the extract (AD + Extract). Gene expression levels of (**a**) filaggrin (**b**) loricrin (**c**) involucrin (**d**) COL1A1 were determined by real-time PCR and normalized to the gene expression of the housekeeping gene RP38. Statistical significances were tested by c) Kruskal-Wallis test with subsequent Dunn’s multiple comparison and (**a**,**b**,**d**) Welch’s ANOVA with subsequent Dunett´s T3 multiple comparison test. *p*-values were marked by asterisks: * *p* < 0.05, ** *p* < 0.01 (*n* = 9–18 stimulations).

**Figure 2:**
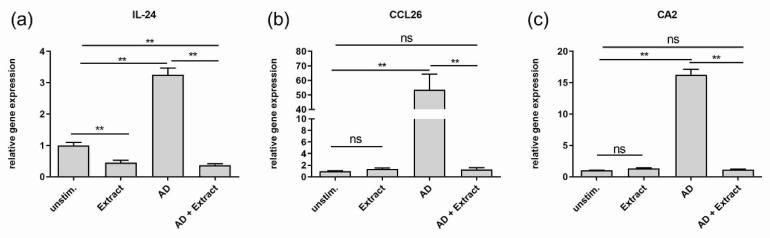
Effect of the plant extract mixture on the gene expression of inflammation marker in an AD-like in vitro model. NHEKs not treated with specific cytokines were used to reflect healthy skin conditions. These cells were left unstimulated (unstim.) or stimulated with the plant extract (Extract) for 20 h. NHEKs were also treated for 20 h with an AD cytokine mix (IL-22, TNF-alpha, IL-13 and IL-4 (each 10 ng/mL)) either in the absence (AD) or in the presence of the extract (AD + Extract). Gene expression levels of (**a**) IL-24 (**b**) CA2 (**c**) CCL26 were determined by real-time PCR and normalized to the gene expression of the housekeeping gene RP38. Statistical significances were tested by (**b**,**c**) Kruskal-Wallis test with subsequent Dunn’s multiple comparison and (**a**) Welch’s ANOVA with subsequent Dunett´s T3 multiple comparison test. *p*-values were marked by asterisks: ** *p* < 0.01 (*n* = 9–18 stimulations).

The effects of the plant extract were determined using either a normal (I) or an AD (II) cell culture in vitro model.

(I) For simulating healthy skin conditions, untreated differentiated normal human keratinocytes (NHEKs) were used to evaluate the effects of the extract in vitro. Stimulation with the extract led to significant two to three-fold higher gene expressions of the epidermal differentiation markers filaggrin and loricrin and the extracellular matrix molecule collagen type I alpha 1 chain (COL1A1) as compared to unstimulated keratinocytes (Figure 1 a,b,d). No significant upregulation of gene expression was observed for involucrin (Figure 1c). Analysis of the inflammation marker revealed a significant downregulation of IL-24 gene expression (Figure 2a) by the extract. No effects of the extract on the already low gene expression of CCL26 and carbonic anhydrase 2 were seen (Figure 2b,c).

(II) To evaluate the effects of the extract on AD in vitro, an AD-like model was generated by incubating NHEKs with an AD-typical cytokine mix consisting of IL-22, TNF-alpha, IL-13 and IL-4 [24]. It could be shown that the cytokine mix was able to mimic skin barrier dysregulation as well as an AD-typical inflammation in vitro. First, the skin barrier dysregulation was represented by the significant downregulation of the gene expression of the epidermal differentiation markers filaggrin and loricrin and, by trend, also involucrin (*p* = 0.069) as compared to untreated keratinocytes, which reflect the healthy skin status (Figure 1a–c). In addition, the gene expression of the extracellular matrix protein COL1A1 was significantly downregulated (Figure 1d).

The AD-typical inflammation induced by the AD cytokine mix was seen by the significant and strong upregulation of the gene expression of IL-24, CCL26 and CA2 in comparison to the gene expression in unstimulated cells (Figure 2).

Adding the extract to the AD-like in vitro model had a strong impact on the gene expression profile of skin barrier molecules as well as inflammation markers. On the one hand, the extract restored the downregulated expression of all skin barrier molecules (Figure 1) and reached at least the height of the expression levels of unstimulated cells, or in the case of filaggrin, loricrin and COL1A1, even a significant two-fold higher level (Figure 1a,b,d). On the other hand, the extract downregulated the gene expression of the inflammation marker CCL26 and CA2 significantly to the level of unstimulated cells (Figure 2b,c) or in case of IL-24, even significantly lower, as shown in Figure 2a.

### 3.3. Penetration Studies

The extract provides high antioxidant and anti-inflammation properties and enhances the skin barrier function. The question arose whether the formulations or parts of it reach the living cells of the epidermis. Representative averaged Raman spectra of verum and placebo creams (Supplementary Figure S1) as well as cream-treated and untreated skin (Supplementary Figure S2) show a large difference in fluorescence intensity characteristic of the verum extract, allowing determination of the penetration depth using the CRM system.

**Figure 3:**
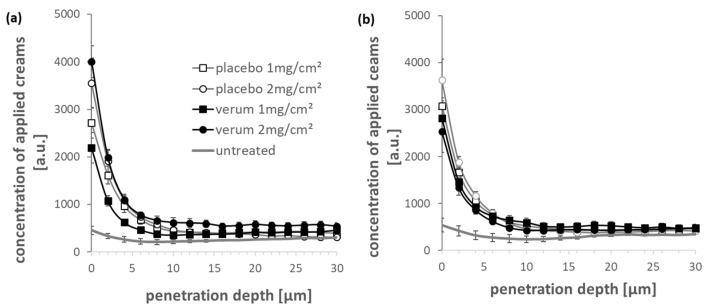
The penetration depth profiles of verum and placebo creams into porcine ear skin obtained after (**a**) 2 and (**b**) 4 h penetration time using CRM compared to untreated skin.

The penetration profiles of the two creams into the skin are shown in Figure 3. For both creams, the concentration of penetrated compounds decreases exponentially from the surface into the skin. There is not enough evidence to show that there is a difference between the verum and the placebo cream in the penetration depth of cream compounds. The typical stratum corneum thickness is approx. 18 µm [29], so compounds from both verum cream and the placebo cream can permeate the entire stratum corneum. The penetration depth of these compounds (both creams) is not significantly related to the penetration time and dosage.

### 3.4. Clinical Study

The absolute values compared with the baseline of all measured items are shown in Table 3.

**Figure 4:**
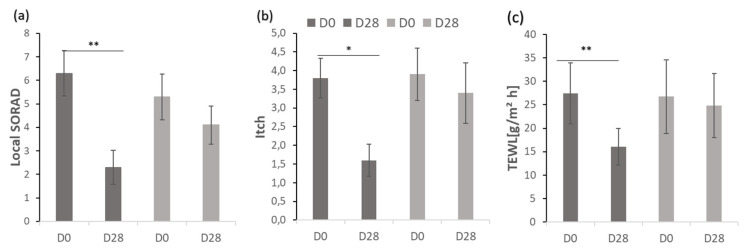
Comparison of (**a**) local SCORAD, (**b**) itch and (**c**) TEWL after four weeks (D28) compared to baseline (D0). Mean ± SEM (*n* = 10); level of significance * *p* < 0.05, ** *p* < 0.01.

The clinical outcomes of local SCORAD, itching and TEWL as a measure of the barrier function are shown in Figure 4.

**Figure 5:**
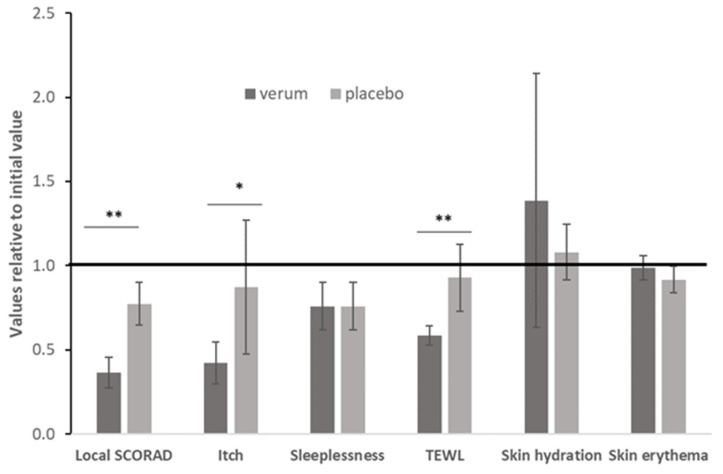
Comparison of the outcomes, assessed as relative value from baseline; level of significance * *p* < 0.05, ** *p* < 0.01.

The relative changes to the initial values are shown in Figure 5. In addition to the before presented parameters, sleeplessness, skin hydration and skin erythema were assessed.

At baseline (0 week), there were no significant differences in the local severity score, itching intensity and TEWL between the symmetrical test areas. After applying verum and placebo cream, the topical SCORAD was reduced by 63.5 and 22.6%, respectively. For the side where verum cream was applied, the difference in local SCORAD before and after treatment was statistically significant (*p* = 0.005, Figure 4). The evaluation of the itching intensity showed that after treatment with verum cream, the visual analogue scale values were significantly reduced (*p* = 0.011, Figure 4). In contrast, the difference before and after the application of the placebo cream was not statistically significant for any of the parameters studied. At 28 days, the TEWL values of the two groups were lower than the baseline. Compared with placebo cream, the difference between before (27.44 ± 6.54, Table 3) and after (16.03 ± 3.92, Table 3) verum cream treatment was more pronounced and statistically different (*p* = 0.005, Figure 4). Taken together, the application of verum cream can significantly reduce the clinical severity score, the intensity of itching and TEWL, but there is no significant difference before and after the application of placebo. At baseline (0 week), there were no significant differences in the erythema, sleeplessness intensity, skin hydration (capacitance) between the symmetrically located test areas. Since the evaluation of insomnia is a systemic assessment of the whole body, it is impossible to compare the effects of the two creams, and the application of two creams does not improve the patient’s sleep quality (Figure 5). Neither cream can improve or reduce skin erythema (Figure 5). Although there was no statistical difference in skin hydration between the two groups before and after treatment, it can be seen from the average and relative values that the application of verum can increase the hydration of the skin while the placebo cannot (Figure 5 and Table 3). In summary, neither verum nor placebo can reduce erythema, improve sleep or increase skin hydration (capacitance) significantly.

## 4. Discussion

As mentioned above, the basic treatment strategy for AD patients is to establish a patient-centered long-term management plan to apply moisturizers to restore the skin barrier while increasing or decreasing the use of anti-inflammatory treatments according to the severity of the disease [36,37].

For patients with mild AD, topical therapy is normally usually used to control inflammation, while for patients with moderate to severe AD, it is necessary to consider whether to use systemic therapy according to the international guideline and the individual situation [13].

Topical corticosteroids and calcineurin inhibitors are the first-line for topical anti-inflammatory treatment, although appropriate intermittent use can avoid the risks such as telangiectasia and skin atrophy [38]. For a specific group of people, such as children, AD patients with facial dermatitis, and patients with frequent relapse, there is no doubt that a non-steroidal topical skin care product which has a certain anti-inflammatory effect and repairs the skin barrier is needed.

In recent years, with the exploration of the pathogenesis of atopic dermatitis, the role of oxidative stress in the occurrence and development of AD has gradually emerged. The hallmark pathological feature of AD is chronic dermatitis of no distinctive type. The activation of inflammatory cells increases the production of free radicals, and excess free radicals can also up-regulate the expression of pro-inflammatory cytokines [39]. At the same time, the change in the antioxidant capacity of AD patients cannot be ignored. A study conducted in preschool-age children to explore the relationship between glutathione-S-transferase polymorphisms and atopic dermatitis risk indicated that the reduction of antioxidant capacity in AD patients might play a role in the pathogenesis [40]. Disorders of the patient’s antioxidant system can affect the progression of the disease from different aspects, including causing itching, enhancing Th2 polarization, and inducing oxidized protein damage in the stratum corneum [41,42].

Given the correlation between oxidative stress and AD pathological factors, antioxidants can be included in the strategy of AD management. In this study, EPR was used to determine the RPF of the extract, and it was concluded that the verum extract has a very high antioxidant capacity of (690 ± 30) × 10^14^ radicals/mg. Most RPF measured for creams so far were far below this value. Nevertheless, already with lower RPF values, strong radical scavenging activities could be found [26,43]. Furthermore, it could be shown that a topically applied ointment containing antioxidants with a high radical protection factor has a number of positive effects on healthy human skin [44,45] and is able to prevent the formation of palmar-plantar erythrodysesthesia which occurs during the chemotherapy of cancer patients [46]. Thus, it is likely that the extract has a certain function of scavenging free radicals in skin lesions which may contribute to the observed effects of the extract. Clearly, more experiments are needed to assess the potential effects of the extract on changes of radicals in skin lesions.

As a barrier organ, the skin is characterized by the ability to selectively allow certain chemicals to pass through the barrier. Most chemicals penetrate the skin through passive diffusion, and few are actively transported. The penetration efficiency of medicinal products is one of the important factors to improve the efficacy of topical preparations. In this study, using the high molecular specificity of the Raman spectrum, according to the chemical composition of the extract, the CRM measurement method was used to evaluate the penetration depth of the extract into porcine ear skin ex vivo. According to our data (Figure 3), the maximum concentration can be detected near the skin surface, and then the concentration gradually decreases with the depth of detection until it stabilizes. Most of the ingredients remain on the skin surface and in the stratum corneum. Nevertheless, some compounds of both creams could permeate the stratum corneum and might reach the metabolically active layers of the viable epidermis. To prove this experimentally, further investigations are necessary. There is no significant difference between the penetration depth of the penetrated compounds from verum and placebo: basically, it is about 20 µm, which is consistent with the lipid order curve of the stratum corneum [47]. There are no significant differences between the penetration depths for 2 and 4 h of penetration time but after 4 h parts of the cream components could be distributed into the surrounding skin compartments and cannot be distinguished anymore from the skin compounds itself. The penetration efficiency of the verum cream components still has room for improvement. Nevertheless, the penetrated amount of the cream components into the stratum corneum could provide a reservoir function for prolonged diffusion of the actives into the viable epidermis. Tracking the penetration of certain active ingredients is possible using Raman spectra analysis [48,49,50]. However, due to the low Raman sensitivity of verum extract components compared to the base cream components, we did not focus on individual compounds in this study. We assume that the penetration depth of the active ingredients of the verum extract corresponds to the penetration depth of the detected verum cream components in the skin. This is very likely, since at a skin depth of 20 µm the fluorescence of the extract is still clearly visible (Figure S2). Nevertheless, this should be verified in further investigations.

In in vitro studies, we confirmed that the extract has anti-inflammatory effects. After adding the extract, the expression of IL-24 in the AD model was significantly reduced, even slightly lower than that of the normal skin model. As a member of the IL-20 cytokine family, IL-24 plays a key role in the pathogenesis of AD [51]. IL-24 down-regulates the expression of filaggrin in keratinocytes, contributing to barrier dysfunction downstream of the IL-13/periostin pathway [51].

At the same time, the induction of CCL26, as a chemotactic factor generated through IL-4 and IL-13 stimulation, was significantly reduced by the extract confirming its anti-inflammatory effect, especially for Th2 immune responses. Similarly, the induction of carbonic anhydrase II (CA2) in the AD in vitro model was also completely blocked by the extract. CA2 is an enzyme commonly expressed in a variety of tissues (such as skin, kidneys, red blood cells), and it plays an important role in maintaining cellular pH. Its expression is significantly upregulated in lesioned AD skin, which is in line with the observation that Th2 cytokines induce its expression in keratinocytes. The enhanced expression of CA2 in AD may contribute to abnormally enhanced pH levels seen in AD skin [52,53]. Compared with the side effects of long-term use of topical corticosteroids on the formation of collagen and vessels, the extract can increase the expression of collagen (COL1A1). In addition, it enhances the expression of skin barrier-related proteins (filaggrin, involucrin and loricrin) supporting the restoration of the skin barrier dysfunction.

The above experimental results were further confirmed by an initial four-week clinical study. The clinical study consisted of a double-blind half-sided comparison in 10 patients. The advantage of the half-sided design is that it basically excludes the individual influences of patients’ psychological and physiological differences on the test results. From the patient’s local SCORAD, it can be seen that compared with placebo cream, the cream with extract significantly improves the symptoms and signs of AD patients. This is attributed to the multiple mechanism of action of the extract: anti-inflammatory/antioxidative effects and skin barrier dysfunction restoration. The improvement of verum on itching degree is also promising. Four weeks after application of the verum, the visual analogue score (VAS) decreased by more than 50%, while the score of the placebo side was basically the same before and after treatment. In patients with AD, pruritus is the most common yet most difficult symptom to control. The itch-scratch cycle leads to bacterial colonization and continuous inflammation, which further aggravates the skin barrier dysfunction. Additionally, the itching has a non-negligible impact on the quality of life and mental health of patients [54]. The effect of the extract on itching is probably mediated by the restoration of the skin barrier function, which reduces the expression of various pro-inflammatory mediators, including neuropeptides [55]. TEWL is a non-invasive measurement method, which can be used to indirectly evaluate the parameters of the skin barrier function [56]. The use of the extract-containing cream significantly reduced the TEWL of AD patients. Skin dysfunction causes the inability to prevent irritants and itching substances (such as allergens) from entering the skin, thus becoming one of the pathogenesis of itch. The hydration of the stratum corneum is related to the dryness of the skin. The drier the skin, the less skin hydration [57]. The water content in the stratum corneum depends mainly on two aspects: (1) bonding of water molecules by the natural moisturizing factor secondary/tertiary structure of keratin inside the corneocytes [58,59] and bonding of water molecules by hydrophilic parts inside the lipid lamellas [60]; and (2) the barrier formed by the orthorhombic lateral packing order of intercellular lipids between corneocytes [61] regulates the TEWL [62]. The decrease of skin hydration indicates the increase of skin TEWL and, at the same time, confirms the skin barrier dysfunction in AD patients [63,64].

During the four weeks of use, patients tolerated verum and placebo well, and no adverse events occurred, which can certify the safety of using extracts. However, the odor and fluidity of the cream preparation still needs to be improved. Since AD is a chronic disease requiring long-term management, the study period of four weeks was too short to determine the safety, furthermore, the small sample size limits the comparison of this extract with other topical drugs, such as topical corticosteroids and tacrolimus ointment.

The extracts used in this experiment are derived from apples, kale and green tea, and the edibility of the sources ensures that the extracts are natural, have very low risk of sensitization, and safety for long-term use because typical plant allergens such as sesquiterpene lactones, terpenes and polyacetylenes are not present in the extract [65]. It is frequently described that herbal, fruit or vegetable extracts are used in topical treatments [66,67,68]. Green tea extract contains high amounts of oligomeric proanthocyanidins such as epigallocatechin 3-gallate (EGCG)—a potent antioxidant with photo-protective properties. However, clinical studies are needed to determine if EGCG has a clinically relevant effect on AD lesions. About the topical application of curly kale and apple extract the reports are rare, but orally administered polyphenol-enriched apple extracts attenuated food allergy in mice [69]. Double blind clinical trials of Applephenon on pediatric patients with atopic dermatitis, and tests using type I allergic model mice suggested that Applephenon might regulate allergic reactions [70]. Wu et al., investigating the therapeutic role of phloretin in mouse model of allergic contact dermatitis found that AD-like symptoms were alleviated and immunopathological effects were reversed [71]. Curly kale extracts rich in carotenoids orally administered have shown to reduce radical formation in skin due to irradiation and protect the collagen in the dermis [72,73]. Furthermore, kaempferol reduces oxidative stress [74] and appears to be an effective topical wound healing agent alone or together with other flavonoids [75,76].To the best of our knowledge, the application of kaempferol extracted from curly kale has not been investigated on skin so far. In the extract used and in the verum formulation of the clinical trial L-arginine is also added. As a constituent of filaggrin, L-arginine can be processed into natural moisturizing factors in the skin [77]. L-arginine can repair the skin barrier from the following two aspects: L-arginine can be hydrolyzed by arginase I into ornithine and urea, which is well known, and can enhance the hydration of the stratum corneum and antimicrobial peptides [78]; L-arginine forms NO through NO synthase (NOS), NO can modulate inflammation, stimulate the proliferation of endothelial cells and re-epithelialization [79,80]. Thus, L-arginine could contribute to improved skin hydration and the reduction of TEWL in the skin of the AD verum group in the clinical study.

The results shown in this paper agree with the findings mentioned in the literature but present for the first time the effect of the combined ingredients from different selected plants in vitro and in vivo after topical application on AD patients.

## 5. Conclusions

In conclusion, the extract applied has strong antioxidant capacity, anti-inflammatory effects, and at the same time increases the expression of skin barrier proteins. Components of the basic formula of the cream containing the extract or part of the extract can penetrate through the stratum corneum but the available experimental data are not sufficient to prove that they could reach other layers of the epidermis. The basic cream formulation still has room for improvement to achieve a higher transdermal penetration efficiency for the incorporated active ingredients. Nevertheless, the clinical study proved that the new extract significantly improves the clinical signs and symptoms in patients with mild to moderate AD. The treatment is well-tolerated, which may provide a new topical skin care product for AD patients.

## Figures and Tables

**Table 1:** Composition of verum and placebo creams.

Ingredients	Extract	DAC Basis Creme
	25.0 g curly kale extract (30% kaempferol flavonoids) 25.0 g green tea extract (60% epigallocatechin gallate) 25.0 g apple extract (15% phlorizin, 5% quercetin flavonoids) 25.0 g l-arginine	4.0 g glycerol monostearate 60 6.0 g cetylalcohol 7.5 g medium-chain trigylcerides (mygliol^®^812, neutral oil) 25.5 g white vaseline (petroleum jelly) 7.0 g macrogol-20-glycerol monostearate 10.0 g propylene glycol 40.0 g purified water
Verum (*w*/*w*)	2%	98%
Placebo (*w*/*w*)	0%	100%

**Table 2:** Primer sequences used in the real-time PCR to determine the gene expression level of AD-relevant genes.

Gene	Forward Primer	Reverse Primer
Carbonic Anhydrase II, CA2	5′-AACAATGGTCATGCTTTCAACG-3′	5′-TGTCCATCAAGTGAACCCCAG-3′
CC-chemokine ligand 26, CCL26,	5′-AATTGAGGCTGAGCCAAAGA-3′	5′-ATCAGGCCCTTCTCAGGTTT-3′
Collagen, type 1, alpha 1, COL1A1	5′-CTGGAAGAGTGGAGAGTACTG	5′-GTCTCCATGTTGCAGAAGAC-3′
Filaggrin, FLG	5′-GGCAAATCCTGAAGAATCCAGATG-3′	5′-GGTAAATTCTCTTTTCTGGTAGACTC-3′
Interleukin-24, IL-24	5′-GTTCCCCAGAAACTGTGGGA-3′	5′-CGAGACGTTCTGCAGAACC-3′
Involucrin, IVL	5′-GGAGGAGGAACAGTCTTGAGG-3′	5′-CTGCCTCAGCCTTACTGTGA-3′
Loricirin, LOR	5′-CTCTCCTCACTCACCCTTCCT-3′	5′-AGGTCTTCACGCAGTCCAC-3‘
Homo sapiens ribosomal protein L38, RPL38	5′-TCAAGGACTTCCTGCTCACA-3′	5′-AAAGGTATCTGCTGCATCGAA-3′

**Table 3:** Clinical and non-invasive bioengineering assessment at baseline (D0) and after four weeks (D28), Mean ± SEM (*n* = 10).

Skin Parameter	Baseline	Day 28
Local SCORAD		
Verum	6.3 ± 1.0	2.3 ± 0.7
Placebo	5.3 ± 1.0	4.1 ± 0.8
Itch		
Verum	3.8 ± 0.5	1.6 ± 1.4
Placebo	3.9 ± 0.7	3.4 ± 0.8
Sleeplessness		
Verum	2.9 ± 0.9	2.2 ± 0.8
Placebo	2.9 ± 0.9	2.5 ± 1.0
TEWL		
Verum	27 ± 7	16 ± 4
Placebo	27 ± 8	25 ± 7
Skin capacitance		
Verum	20 ± 5	28 ± 5
Placebo	23 ± 5	25 ± 5
Skin erythema		
Verum	340 ± 30	340 ± 40
Placebo	340 ± 40	310 ± 20

## Data Availability

All of the data is contained within the article and the supplementary materials.

## Supplementary Materials

The following supporting information can be downloaded at: https://www.mdpi.com/article/10.3390/antiox11061071/s1, Figure S1: Representative averaged Raman spectra of verum (a) and placebo (b) creams; Figure S2: Representative averaged Raman spectra of untreated skin (left column), placebo cream-treated skin (middle column) and verum cream-treated skin (right column) recorded at different exemplary skin depths (2, 8, 16 and 22 µm). Stratum corneum thickness is 18 µm after 2 hours penetration time.
