# Supplementary material for: Skin Care Product Rich in Antioxidants and Anti-Inflammatory Natural Compounds Reduces Itching and Inflammation in the Skin of Atopic Dermatitis Patients
Source: Antioxidants (Basel). 2022 May 28;11(6):1071. doi: 10.3390/antiox11061071 (PMC9219975; doi:10.3390/antiox11061071)
Supplement: Supplementary file 1 [file antioxidants-11-01071-s001.zip › antioxidants-1694871-supplementary.pdf]

Supplementary Material

a) Verum b) Placebo

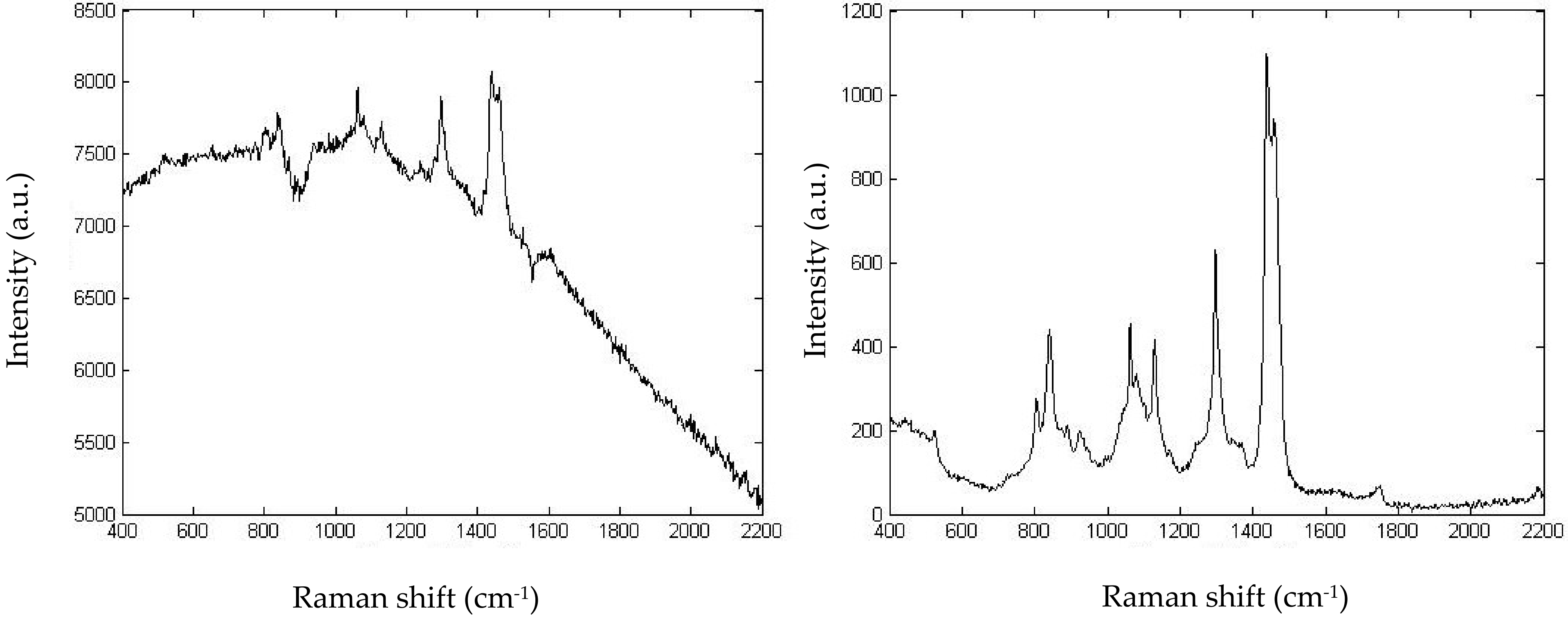

Figure S1. Representative averaged Raman spectra of verum a) and placebo b) creams

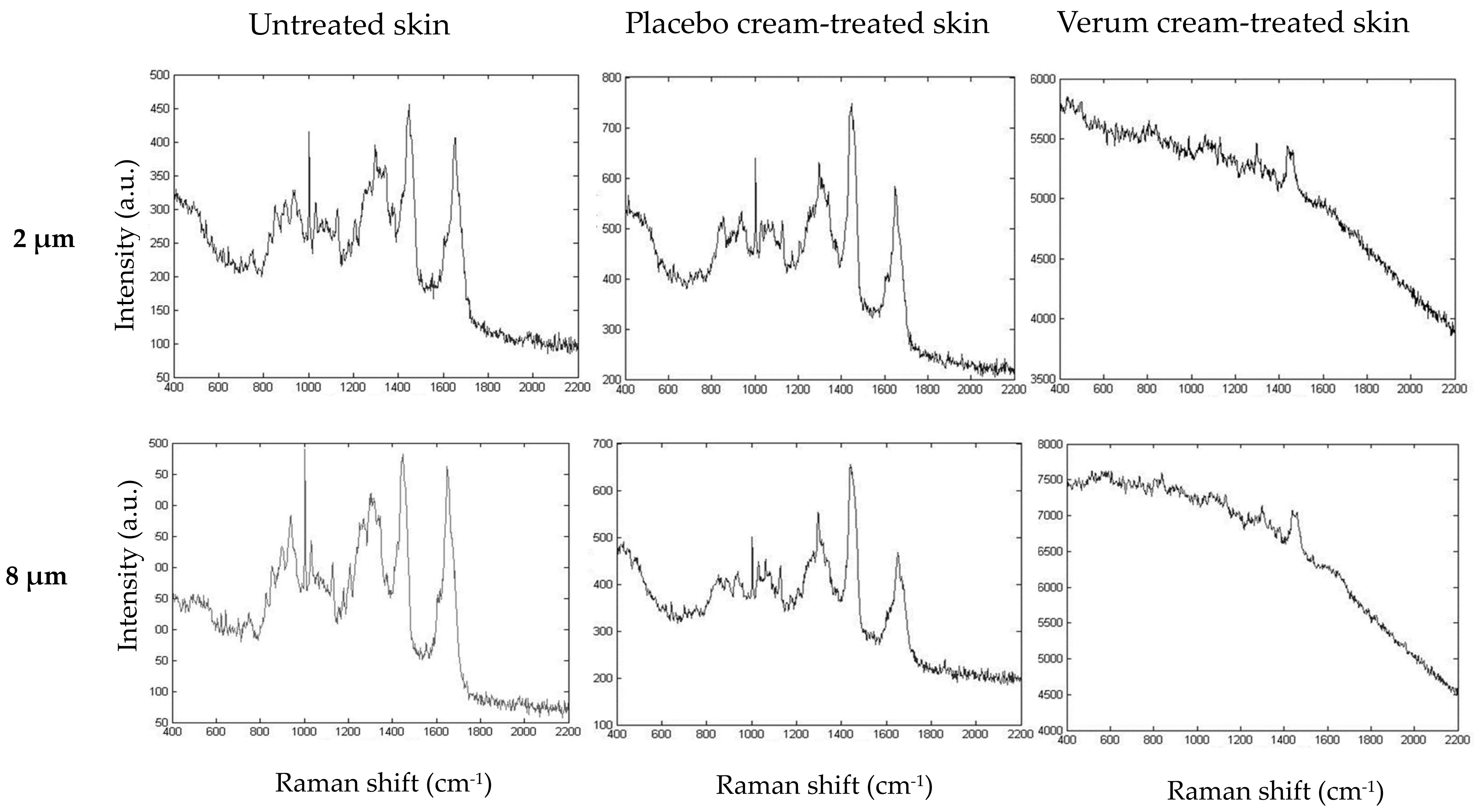
Untreated skin
Placebo cream-treated skin
Verum cream-treated skin
2 μm
8 μm
Intensity (a.u.)
Intensity (a.u.)
Raman shift (cm-1)
Raman shift (cm-1)
Raman shift (cm-1)

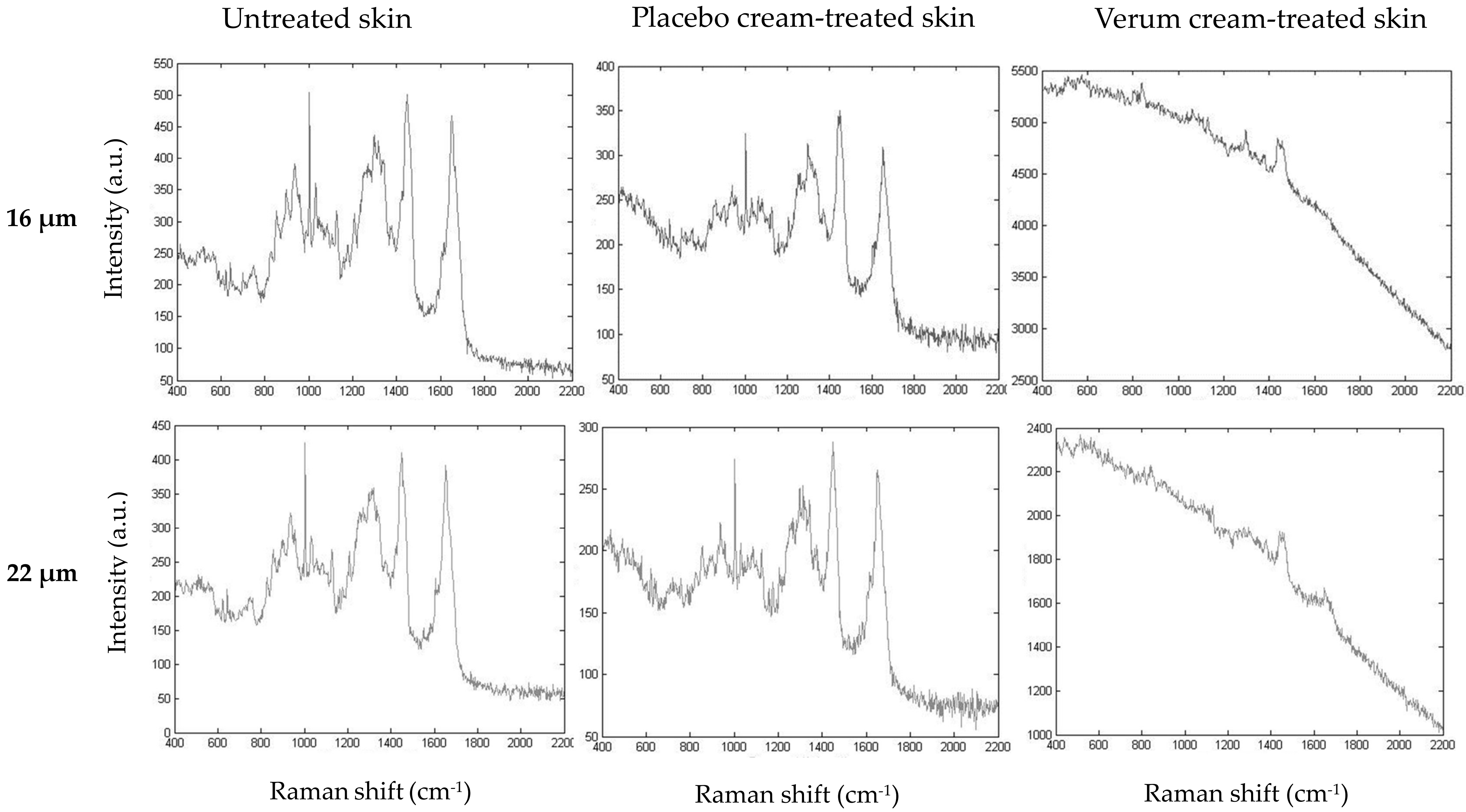
Untreated skin
Placebo cream-treated skin
Verum cream-treated skin
16 μm
22 μm
Intensity (a.u.)
Intensity (a.u.)
Raman shift (cm-1)
Raman shift (cm-1)
Raman shift (cm-1)

Figure S2. Representative averaged Raman spectra of untreated skin (left column), placebo cream-treated skin (middle column) and verum cream-treated skin (right column) recorded at different exemplary skin depths (2, 8, 16 and 22 μm). Stratum corneum thickness is 18 μm after 2 hours penetration time.
